# Supplementary material for: Machine learning models highlight environmental and genetic factors associated with the Arabidopsis circadian clock
Source: Nat Commun. 2025 Aug 5;16:7223. doi: 10.1038/s41467-025-62196-w (PMC12325936; doi:10.1038/s41467-025-62196-w)
Supplement: Supplementary file 1 — Supplementary Information [file 41467_2025_62196_MOESM1_ESM.pdf]

Gene ID	Name	meta2d Phase	meta2d Period	meta2d Q-value
AT1G01060	*LHY*	23.69	24.10	2.25E-06
AT1G22770	*GI*	9.44	23.95	1.29E-05
AT2G21070	*FIO1*	15.02	18.07	0.07
AT2G25930	*ELF3*	15.92	22.85	1.54E-05
AT2G31870	*TEJ*	13.95	23.09	0.004
AT2G46790	*PRR9*	5.83	24.17	0.0002
AT2G46830	*CCA1*	0.88	24.40	1.80E-05
AT3G20810	*JMJD5*	15.17	23.15	0.0006
AT3G22380	*TIC*	15.85	22.29	0.005
AT3G46640	*PCL1*	12.46	23.81	0.006
AT4G39620	*EMB2453*	15.24	22.24	0.04
AT5G02810	*PRR7*	7.48	24.17	1.34E-05
AT5G42900	*COR27*	13.11	23.95	0.0001
AT5G57360	*ZTL*	13.35	24.02	0.0005
AT5G59570	*BOA*	14.98	24.25	0.0007
AT5G60100	*PRR3*	13.77	23.15	0.0002
AT5G61380	*TOC1*	13.75	23.28	0.0001

Supplementary Table 1: List of 17 clock genes selected based on prior biological knowledge and the fact they are consistently expressed across the training data. Includes approximations for phase period length and rhythmicity Q-values as determined using MetaCycle[1] within the continuous-light (LL) time-course by *Romanowski et al.*[2]

Model	*N* gene features	Parameters	Tuning scheme	Tuned MAE (mins)
ZeitZeiger	89	*sumabsv* (regularization): 5<br>N sparse principal-components: 2	LOO cross-validation	52.9<br>(± 42.0)
PLSR ensemble	233<br>(out of 100 sets)	N latent variables: 7	5-fold cross-validation	14.8<br>(± 14.5)
Taufisher	22<br>(484 gene-pair features)	N principal-components: 3	LOO cross-validation	88.9<br>(± 63.1)
MolecularTimetable<br>**(WSN)**	189	N phases: 144<br>Pearson's *r* threshold > 0.89<br>Variation threshold > 0.15<br>Within-study-normalization	Training data	47.1<br>(± 63.2)
TimeSignatR<br>**(WSN)**	167	*α* (regularization): : 0.25<br>λ (shrinkage): exp(-2.0)<br>Within-study-normalization	LOO cross-validation	14.3<br>(± 11.4)

Supplementary Table 2: List of previously published circadian time (CT) predictors trained to compare with ChronoGauge including ZeitZeiger[3], partial-least-squares-regression[4] (PLSR as an ensemble), Taufisher[5], MolecularTimetable[6] and TimeSignatR[7]. Includes *N* gene features used as an input, the parameters & normalization techniques selected, the methods used to find said parameters and the mean absolute-errors (MAEs) of the tuned models across training/cross-validation data. We note MolecularTimetable was tuned without cross-validation because cosine wave fitting may not fit appropriately across folds.
LOO: Leave-one-out, **WSN:** within-study-normalization

RNA-seq test benchmark data						
Model	MdAE (mins)		MAE (mins)		*r*	
	Non-corrected	Combat-seq	Non-corrected	Combat-seq	Non-corrected	Combat-seq
ChronoGauge (x100)	**20.6**	27.5	44.0 (± 47.6)	**43.2 (± 40.7)**	0.990	**0.992**
ZeitZeiger	46.3	32.9	73.8 (± 69.2)	48.9 (± 46.9)	0.976	0.991
Taufisher	90.0	60.0	105.5 (± 68.0)	92.1 (± 57.0)	0.985	0.991
PLSR (x100)	38.4	37.5	59.8 (± 49.7)	48.2 (± 39.4)	0.985	0.991
MolecularTimetable	50.0	60.0	54.8 (± 47.0)	60.3 (± 45.6)	0.989	0.988
TimeSignatR	60.2	45.5	65.2 (± 46.9)	56.0 (± 46.4)	0.983	0.987

Supplementary Table 3: Full list of evaluation metrics for each model's circadian time (CT) estimates in a hold-out RNA-seq set[8–13] (*N* samples = 58) using both non-corrected and Combat-seq[14] corrected expression values for training and testing. Includes median-absolute-error (MdAE), mean-absolute-error (MAE) and Pearson correlation coefficient (*r*). Parentheses denote standard-deviation of absolute-errors. Top scores for each metric listed in **bold**.

x100: ensemble of 100 sub-predictors, **PLSR**: Partial-least-squares-regression

ATH1 microarray benchmark test						
Model	MdAE (mins)		MAE (mins)		*r*	
	Non-corrected	Combat-seq	Non-corrected	Combat-seq	Non-corrected	Combat-seq
ChronoGauge (x100)	**46.1**	53.5	**61.8 (± 50.2)**	73.0 (± 57.0)	**0.984**	0.982
ZeitZeiger	77.9	118.1	123.8 (± 103.7)	48.9 (± 127.4)	0.932	0.902
Taufisher	120.0	60.0	116.7 (± 93.6)	97.8 (± 74.0)	0.976	0.983
PLSR (x100)	83.2	98.1	105.3 (± 77.6)	124.0 (± 107.9)	0.965	0.942
MolecularTimetable	70.0	60.0	64.7 (± 44.3)	71.8 (± 50.9)	**0.984**	0.980
TimeSignatR	75.3	69.8	82.3 (± 67.9)	77.9 (± 64.6)	0.971	0.903

Supplementary Table 4: Full list of evaluation metrics for each model's circadian time (CT) estimates in a hold-out ATH1 microarray set[15–18] (*N* samples = 73) using both non-corrected and Combat-seq[14] corrected expression values for training. Microarray test expression values were not batch corrected. Includes median-absolute-error (MdAE), mean-absolute-error (MAE) and Pearson correlation coefficient (*r*). Parentheses denote standard-deviation of absolute-errors. Top scores for each metric listed in **bold**.

x100: ensemble of 100 sub-predictors, **PLSR**: Partial-least-squares-regression

**AraGene microarray benchmark test**						
Model	MdAE (mins)		MAE (mins)		*r*	
	Non-corrected	Combat-seq	Non-corrected	Combat-seq	Non-corrected	Combat-seq
ChronoGauge (x100)	**74.8**	96.9	**61.8 (± 50.2)**	73.0 (± 57.0)	**0.983**	0.974
ZeitZeiger	122.0	109.4	160.3 (± 156.6)	143.2 (± 110.8)	0.911	0.951
Taufisher	120.0	120.0	156.7 (± 113.8)	150.0 (± 92.7)	0.937	0.963
PLSR (x100)	85.5	103.5	128.6 (± 99.7)	128.6 (± 91.9)	0.957	0.964
MolecularTimetable	100.0	110.0	118.3 (± 78.8)	122.9 (± 71.6)	0.971	0.977
TimeSignatR	116.0	104.2	126.2 (± 86.4)	119.1 (± 79.0)	0.959	0.949

Supplementary Table 5: Full list of evaluation metrics for each model's circadian time (CT) estimates in a hold-out AraGene microarray set[19] (*N* samples = 72) using both non-corrected and Combat-seq corrected expression values for training. Microarray test expression values were not batch corrected. Includes median-absolute-error (MdAE), mean-absolute-error (MAE) and Pearson correlation coefficient (*r*). Parentheses denote standard-deviation of absolute-errors. Top scores for each metric listed in **bold**.

x100: ensemble of 100 sub-predictors, **PLSR**: Partial-least-squares-regression

Test set	Experiment	Entrainment photoperiod	Sampling photoperiod	MdAE (mins)
RNA-seq	*Rugnone et al.*[8] *(N = 18)*	16:8	16:8	15.3 (± 16.5)
	Miller et al.[9] *(N = 3)*	16:8	16:8	66.9 (± 57.5)
	Takahasi et al.[10] *(N = 12)*	12:12	LL	72.6 (± 39.9)
	Ezer et al.[11] *(N = 8)*	8:16	8:16	111.8 (± 58.8)
	Graf et al.[12] *(N = 6)*	12:12	12:12	9.4 (± 4.2)
	Dubois et al[13] *(N = 12)*	16:8	16:8	16.3 (± 15.0)
Microarray ATH1	*Edwards et al.*[15] *(N = 13)*	12:12	LL	77.6 (± 57.8)
	Covington et al.[16] *(N = 12)*	12:12	LL	36.4 (± 60.5)
	Michael et al.[17] *(Col-0, N = 6)*	16:8	16:8	34.1 (± 21.8)
	Michael et al.[17] *(Col-0, N = 6)*	8:16	8:16	79.1 (± 48.8)
	Michael et al.[17] *(Ler, N = 6)*	8:16	8:16	100.2 (± 63.7)
	Espinoza et al.[18] *(N = 15)*	16:8	LL	43.3 (± 32.5)
	Espinoza et al.[18] *(N = 15)*	16:8	16:8	43.0 (± 18.0)
Microarray AraGene	*Endo et al.*[19] *(N = 36)*	16:8	16:8	49.4 (± 56.0)
	Endo et al.[19] *(N = 36)*	8:16	8:16	104.0 (± 73.2)

Supplementary Table 6: Full list of median-absolute-errors (MdAEs) across individual transcriptome experiments within each test set. Also listed are photoperiod conditions for entrainment and sampling. Parentheses denote standard-deviation. Italicized text refers to authors of published data.

LL: continuous-light, **16:8**: long-day conditions, **12:12:** neutral-day conditions, **8:16:** short-day conditions

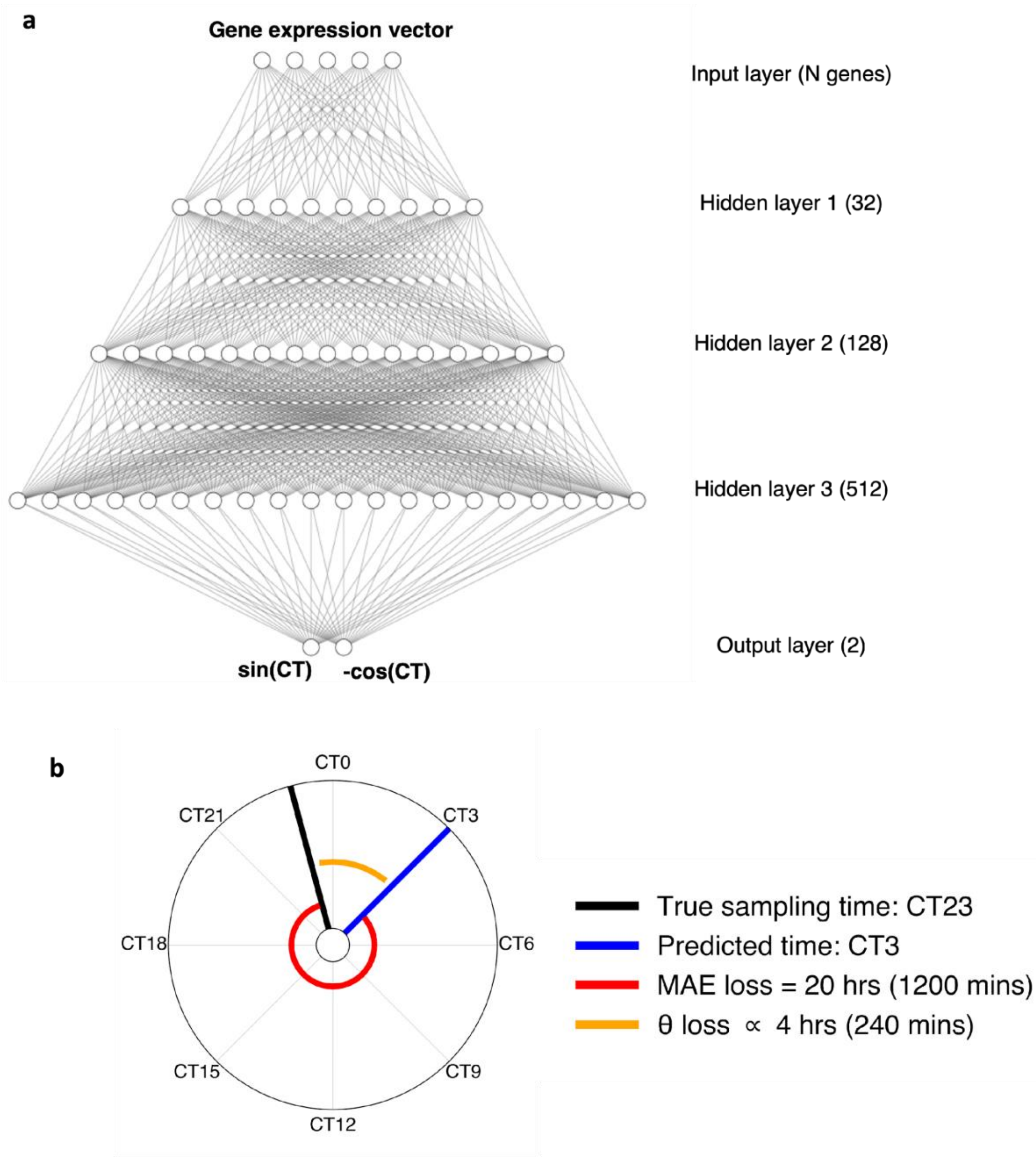

Supplementary Figure 1: Neural network architecture used by ChronoGauge based on a previously published model by *Gardiner et al*.[20]. **a** Multi-layer-perceptron (MLP) composed of 3 hidden layers that outputs the circadian time (CT) as sine and cosine values. **b** Justification of using θ (angle; orange) between true CT at CT23 (black) and predicted CT at CT3 as a loss function compared with mean-absolute-error (MAE) (red). A MAE loss does not consider the circular nature of the CT, while the θ loss does.

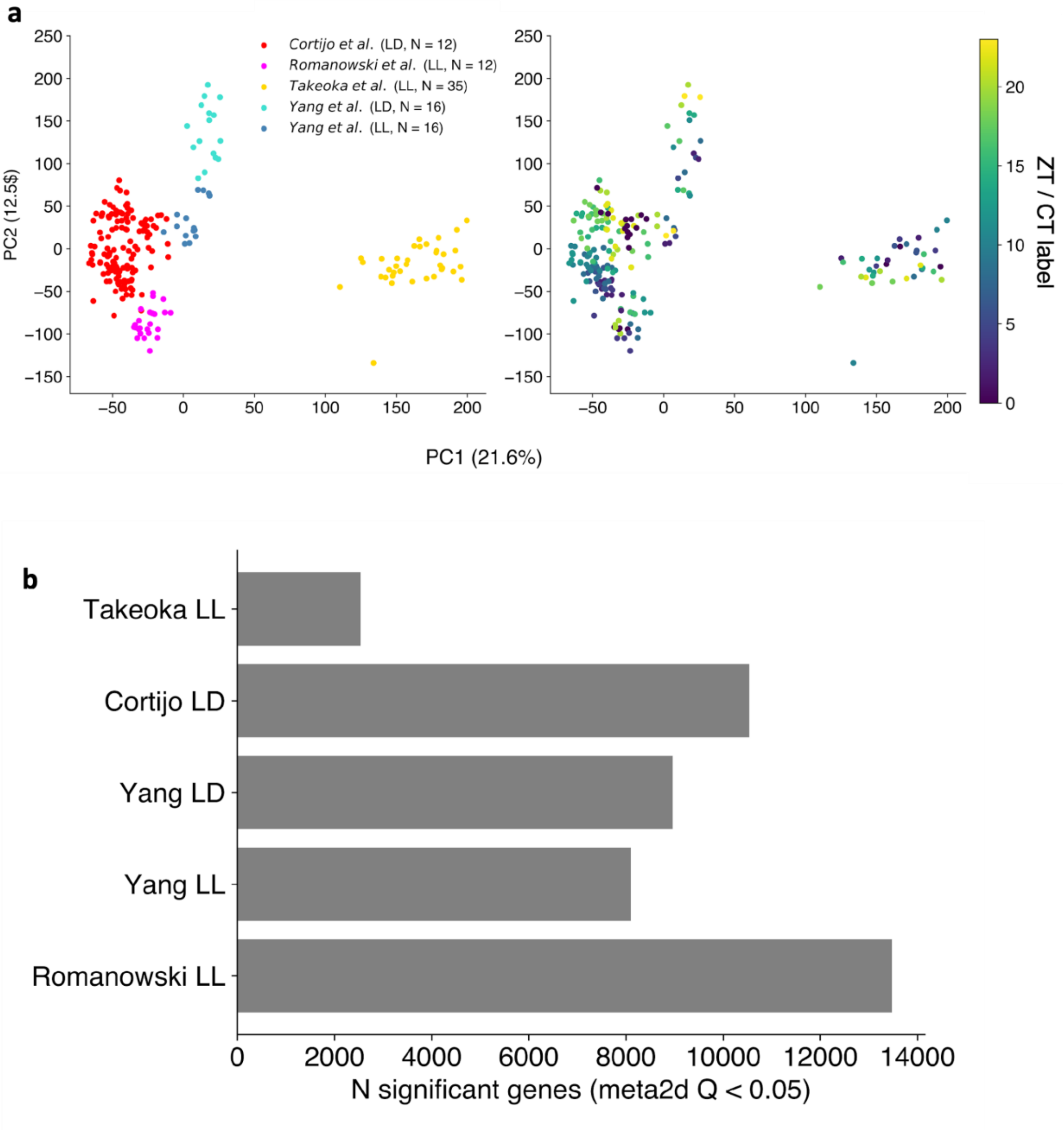

Supplementary Figure 2: Appraisal of the suitability of time-course RNA-seq datasets[2,21–23] harvested under either continuous-light (LL) or a light-dark cycle (LD) for training. **a** variation of proposed test samples labelled by experimental group (left) and circadian time (CT) or zeitgeber time (ZT). **b** Number of genes called as significantly rhythmic across training datasets based on results from MetaCycle[1] (meta2d Q < 0.05). Figure source data provided in Source Data file.

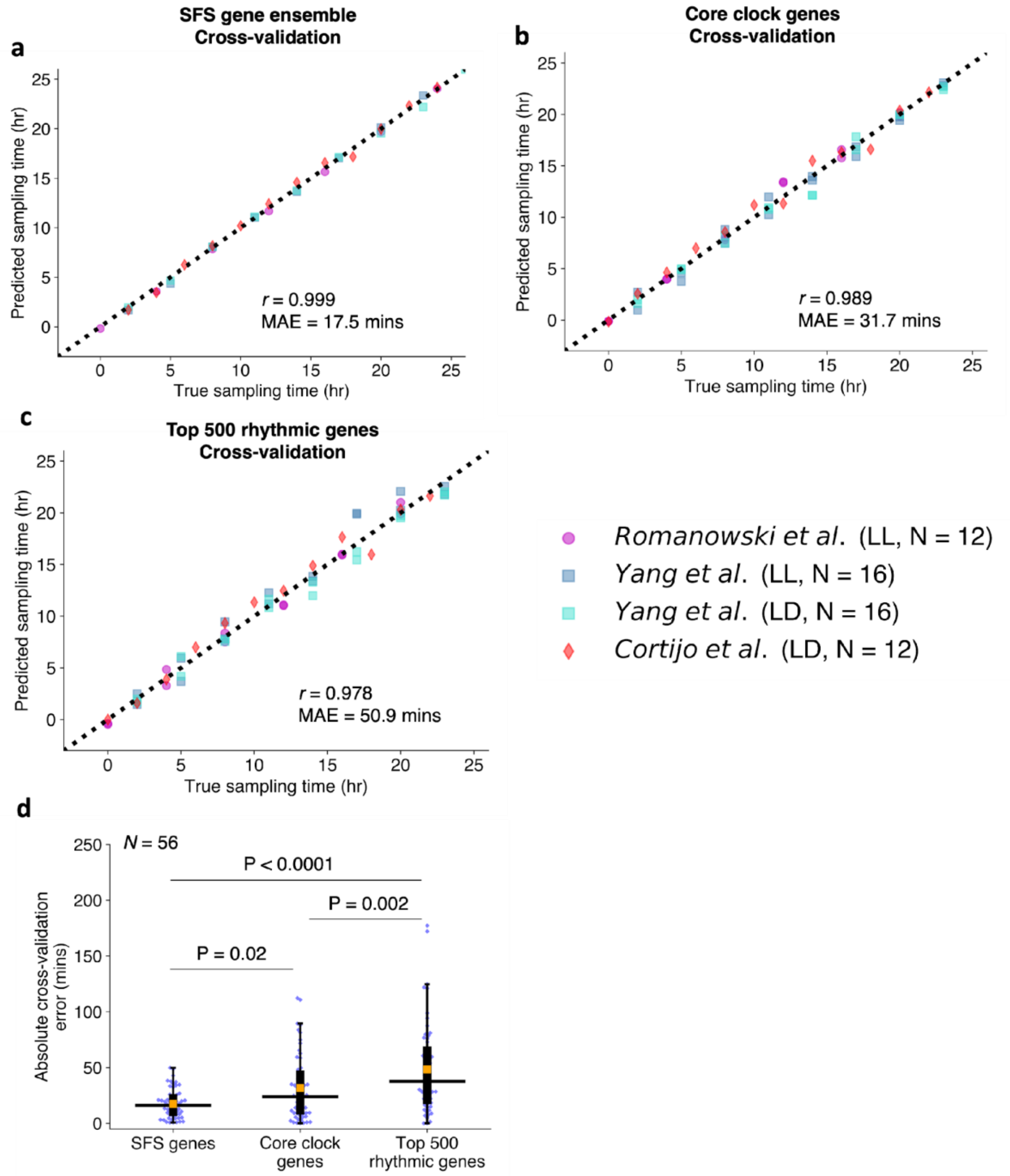

Supplementary Figure 3: Evaluation of tuned neural network (NN) models across 5-fold cross-validation for model selection using a combination of four separate time-course experiments[2,22,23] as a training dataset. Pearson correlation coefficients (*r)* and mean-absolute-errors (MAE) of circadian time (CT) estimates made for **a** using the sequentially selected feature (SFS) ensemble of 100 sub-predictors, **b** a NN trained using 17 core clock genes as features and **c** a NN trained using the top 500 rhythmic genes as determined by meta2d Q-values as features. **d** Comparison of CT absolute-errors (mins) across 5-fold cross-validation. Boxplot properties include: centre line = median-absolute-error (MdAE), orange box = mean-absolute-error (MAE), box limits = interquartile range (IQR), whiskers = 1.5 x IQR, blue points = error of individual samples. MdAEs compared across each NN-model using a two-tailed Wilcoxon signed-rank test with a Bonferroni adjusted P-values. Experiments harvested under either continuous-light (LL) or diurnal/light-dark (LD) conditions are listed. Figure source data provided in Source Data file.

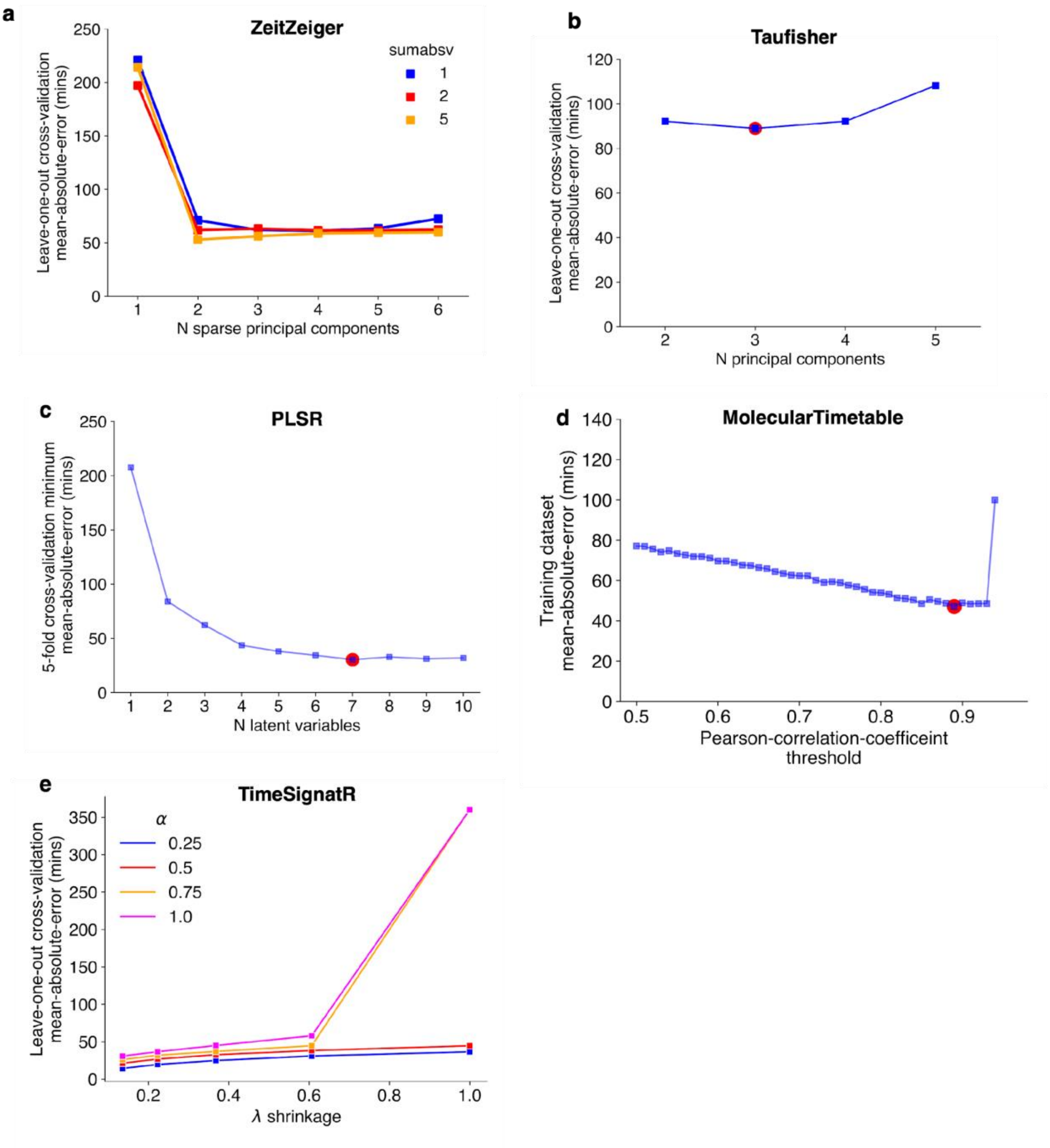

Supplemental Figure 4: Hyperparameter optimization of different circadian time (CT) estimation models using mean-absolute-error (MAE) across the same four time-course experiments[2,22,23] used to train ChronoGauge. **a,** Optimization of sumabsv (regularization factor) and *N* sparse principal components used for ZeitZeiger[3] using leave-one-out (LOO) cross-validation. **b,** Identification of optimal (red circle) *N* principal components used for Taufisher[5] using LOO cross-validation. **c,** Identification of the optimal (red circle) *N* latent variables used by partial-least-squares-regression[4] (PLSR) based on the minimum 5-fold cross-validation MAE displayed across a recursive feature elimination of 500 - 5 genes features. **d**, Identification of the optimal (red circle) Pearson correlation coefficient threshold (*r*) used to select time-indicating genes for MolecularTimetable[6] across the training data. **e,** Identification of the optimal α (regularization factor) and γ (shrinkage factor) used for TimeSignatuR[7] using LOO cross-validation. Figure source data provided in Source Data file.

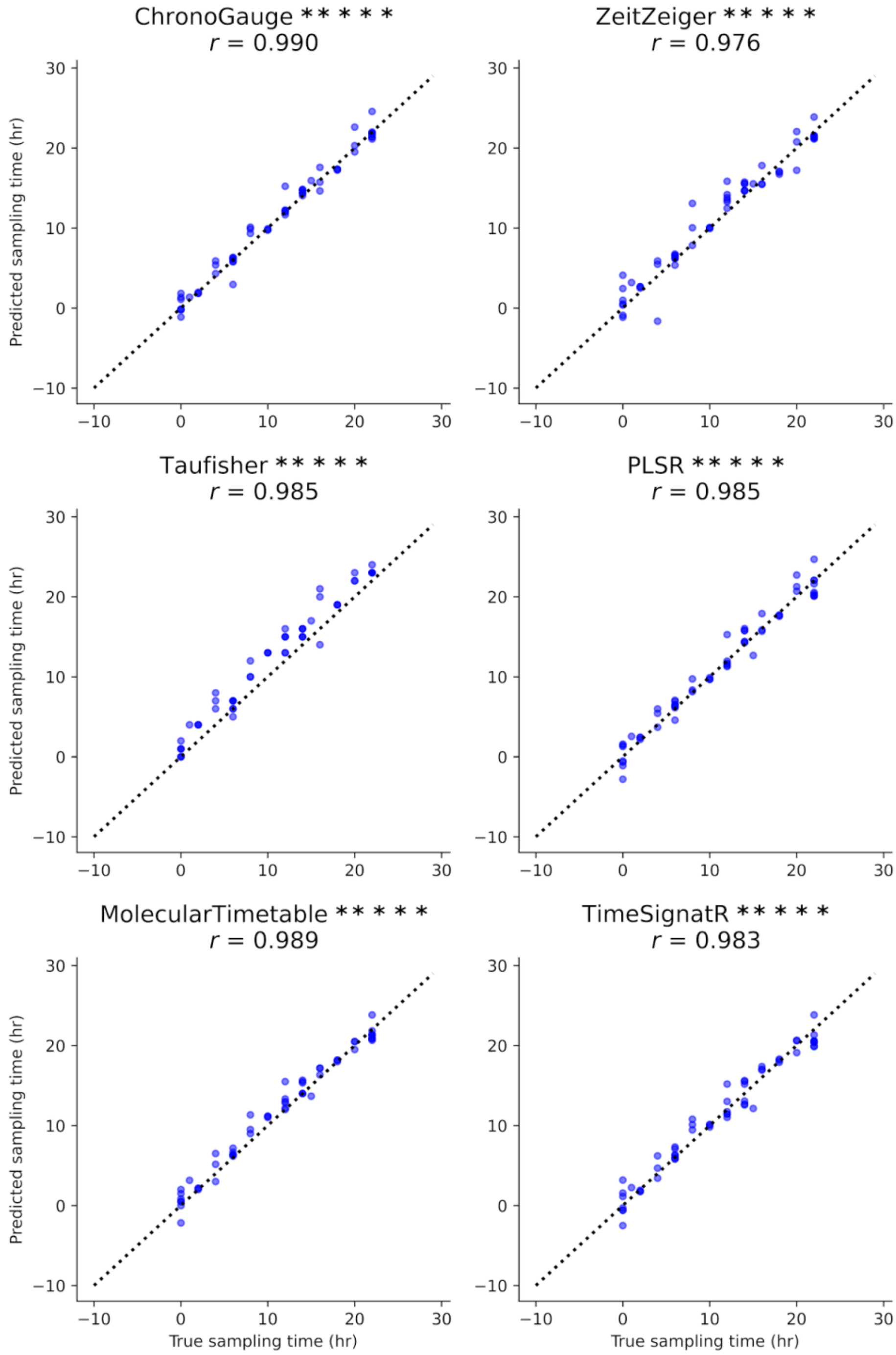

Supplemental Figure 5: Correlation of RNA-seq test samples[8–13] (*N* = 58) true harvesting time labels compared with the circadian time (CT) estimates across different models. CT estimations were adjusted to account for the 24-hour modulus. Pearson correlation coefficients (*r)* and P-values shown. P-values adjusted using Bonferroni method. ***** P < 0.00001. Figure source data with actual P-values provided in Source Data file.

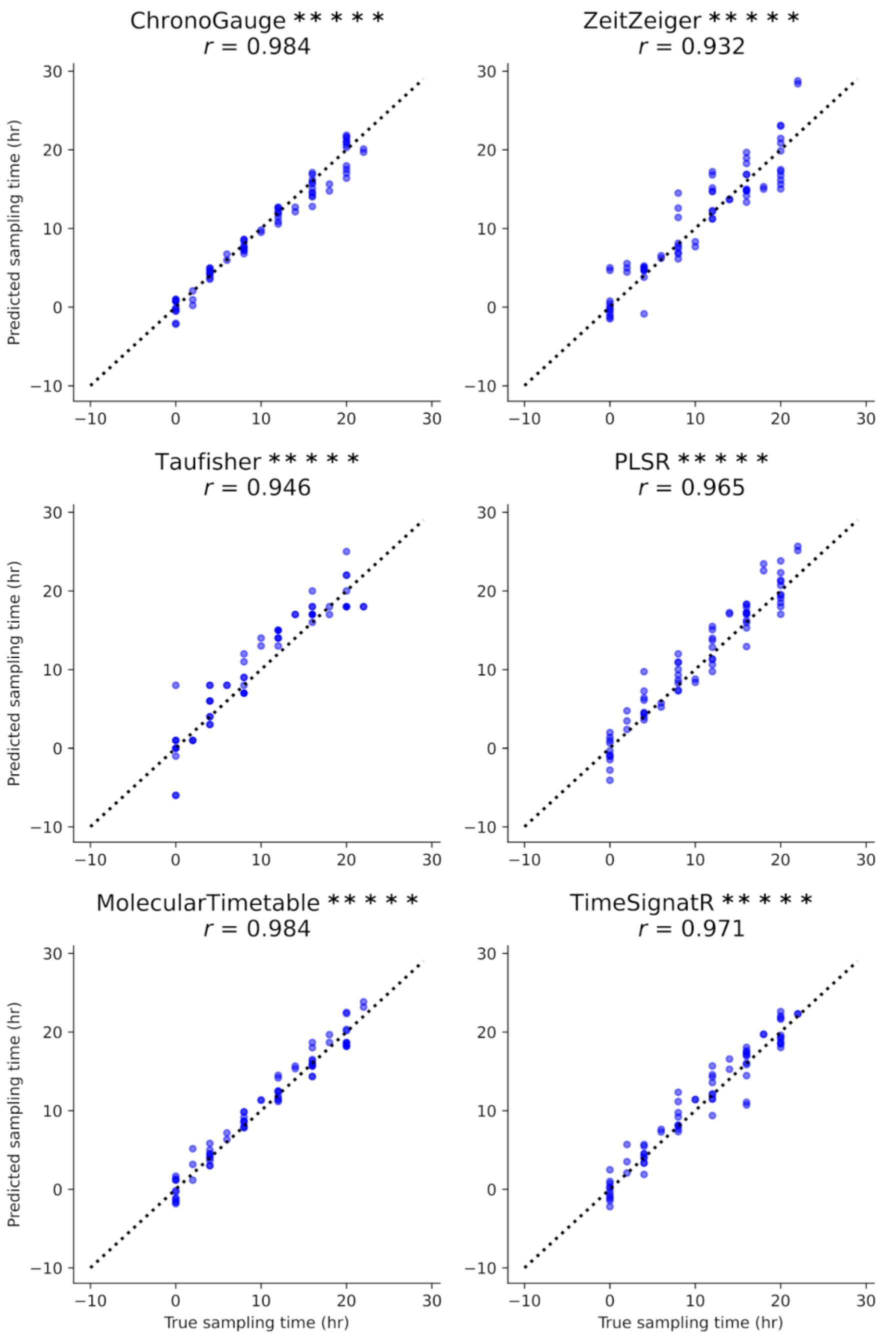

Supplemental Figure 6: Correlation of microarray ATH1 test samples[15–18] (*N* = 73) true harvesting time labels compared with the circadian time (CT) estimates across different models. CT estimations were adjusted to account for the 24-hour modulus. Pearson correlation coefficients (*r*) and P-values shown. P-values adjusted using Bonferroni method. ***** P < 0.00001. Figure source data with actual P-values provided in Source Data file.

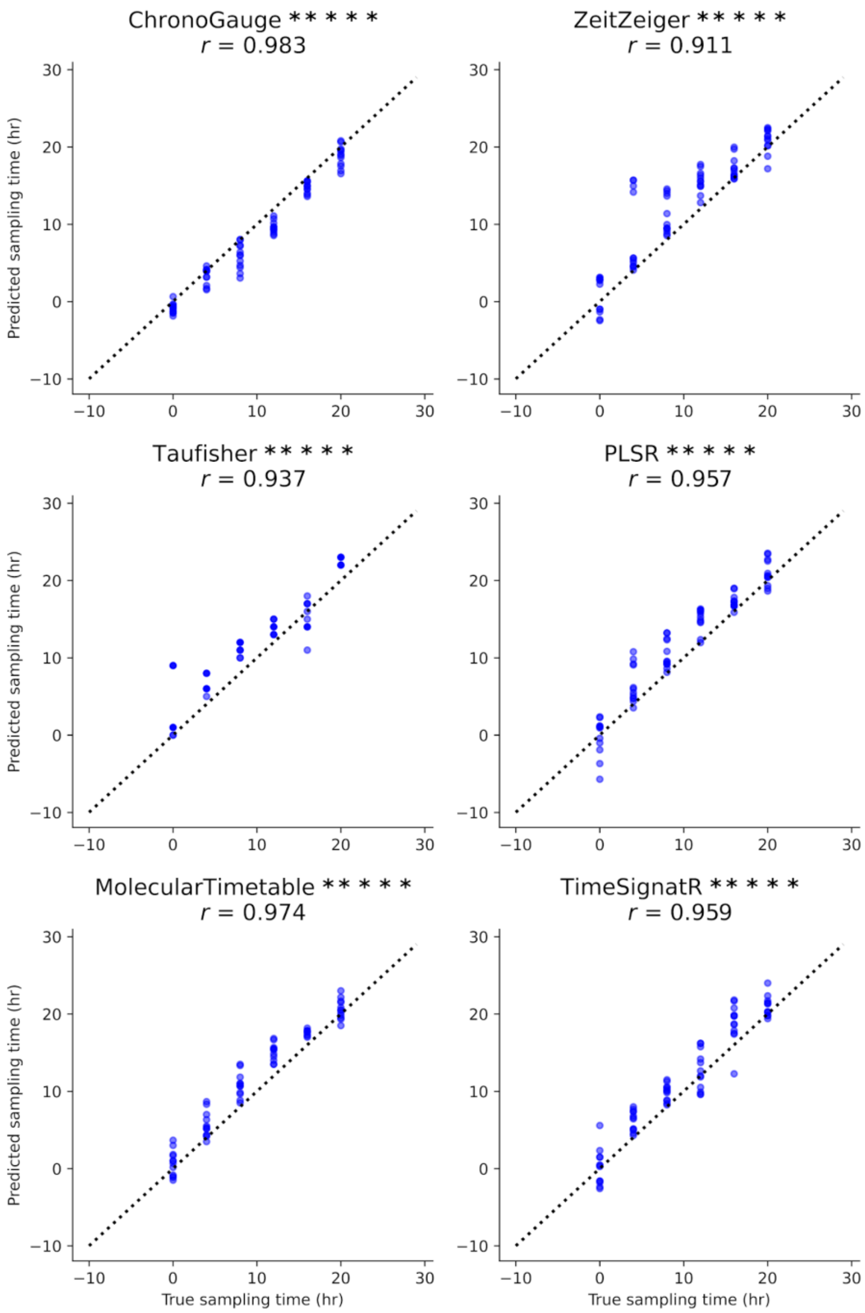

Supplemental Figure 7: Correlation of microarray AraGene test samples[19] ($N = 72$) true harvesting time labels compared with the circadian time (CT) estimates across different models. CT estimations were adjusted to account for the 24-hour modulus. Pearson correlation coefficients (*r)* and P-values shown. P-values adjusted using Bonferroni method. ***** P < 0.00001. Figure source data with actual P-values provided in Source Data file.

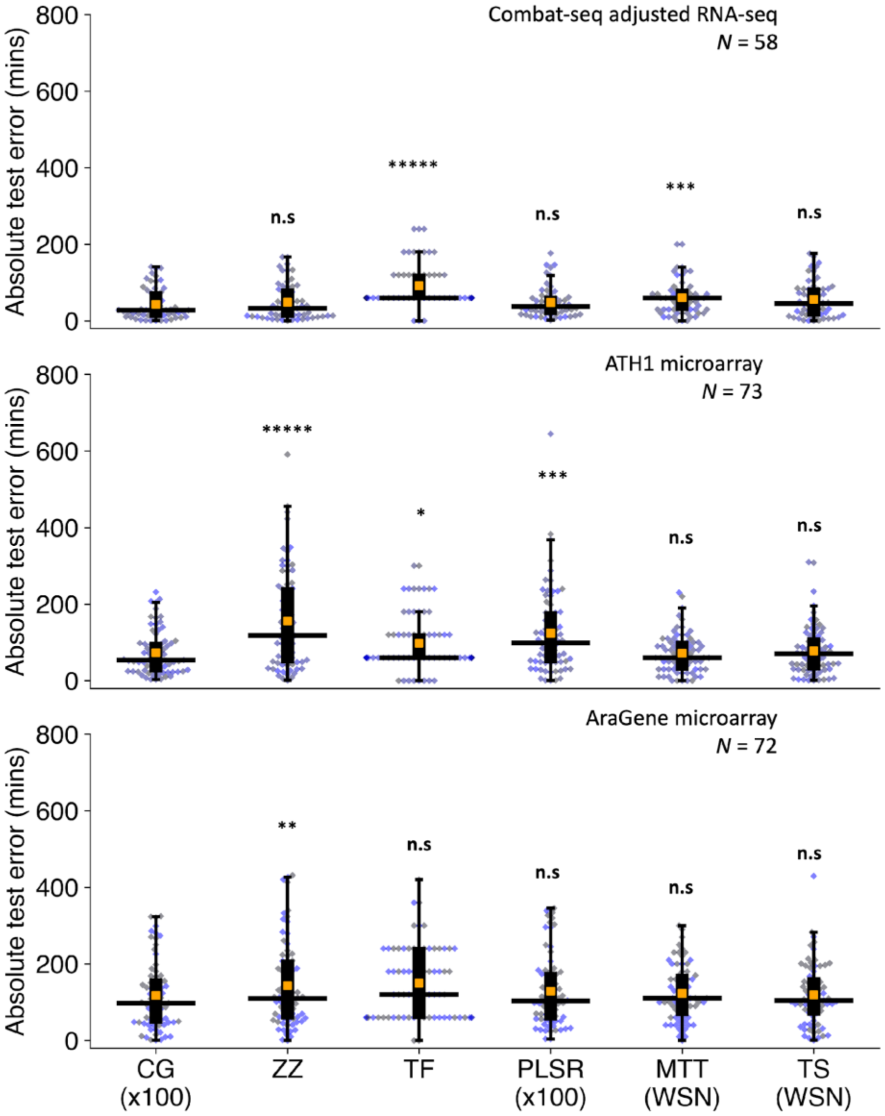

Supplementary Figure 8: Using Combat-seq adjusted RNA-seq expression as an input, comparison of hold-out test set absolute errors between ChronoGauge ensemble (CG x100) and competing circadian time (CT) estimation methods including ZeitZeiger[3] (ZZ), Taufisher[5] (TF), partial-least-squares-regression[4] ensemble (PLSR x100), MolecularTimetable[6] (MTT) and TimeSignatR[7] (TS). Boxplot properties include: centre line = median-absolute-error (MdAE), orange box = mean-absolute-error (MAE), box limits = interquartile range (IQR), whiskers = 1.5 x IQR, blue points = error of individual samples. Comparison includes a Combat-seq[14] adjusted RNA-seq set[8–13] (*N* samples = 58), an unadjusted ATH1 microarray set[15–18] (*N* samples = 73) and an unadjusted AraGene microarray set[19] (*N* samples = 72). Methods using within-study-normalization (WSN) listed. Microarray set signals were not adjusted. Bonferroni-adjusted P-values shown from a Wilcoxon signed-rank test. n.s no significance, * $P < 0.05$, ** $P < 0.01$, *** $P < 0.001$, **** $P < 0.0001$, ***** $P < 0.00001$. Figure source data with actual P-values provided in Source Data file.

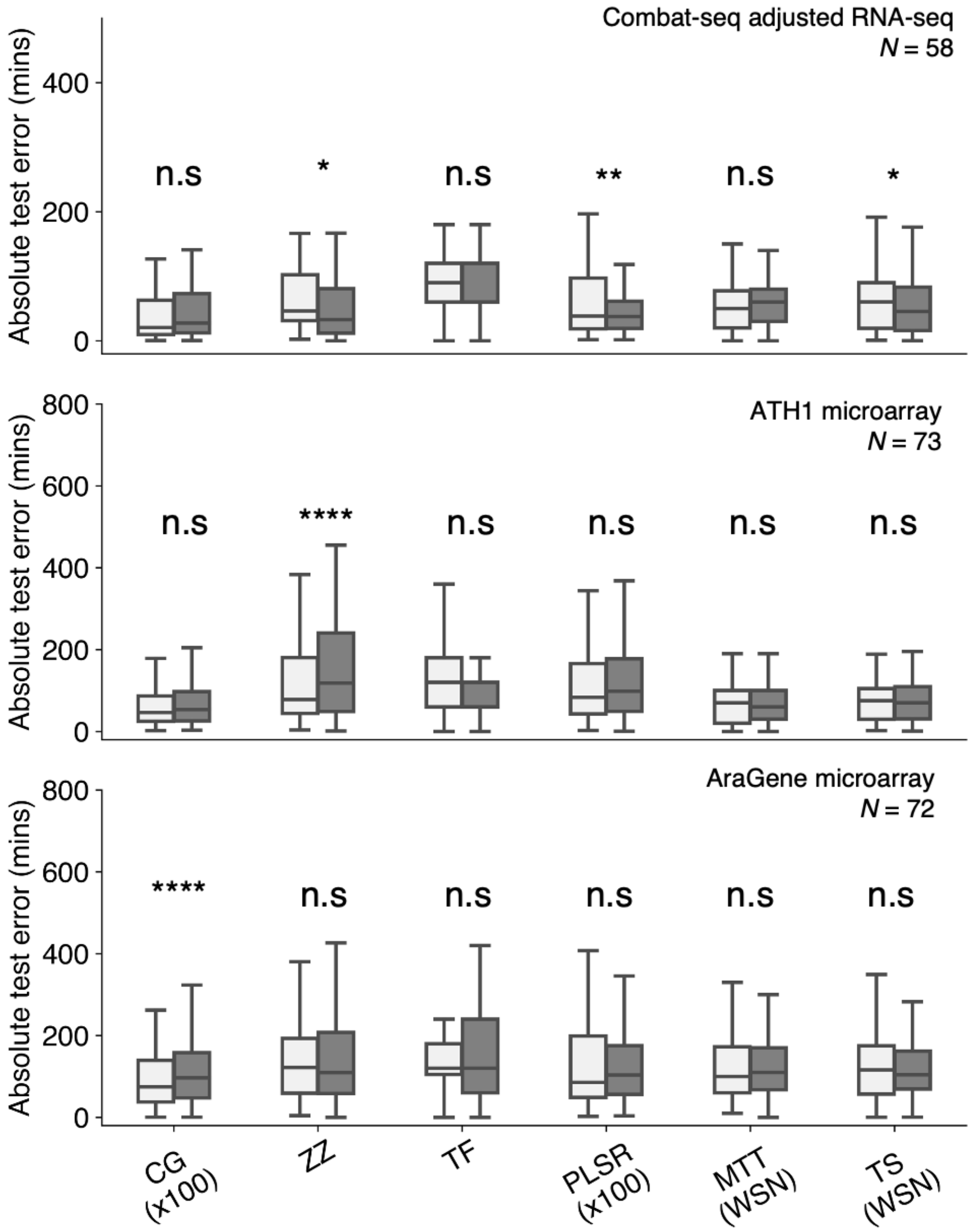

Supplementary Figure 9: For each circadian time (CT) estimation model, comparison of CT estimation absolute errors when fit to unadjusted (white) and Combat-Seq[14] adjusted (gray) gene expression data. Includes ChronoGauge ensemble (CG x100), ZeitZeiger[3] (ZZ), Taufisher[5] (TF), partial-least-squares-regression[4] ensemble (PLSR x100), MolecularTimetable[6] (MTT) and TimeSignatR[7] (TS). Comparison includes a RNA-seq set[8–13] (*N* samples = 58), an ATH1 microarray set[15–18] (*N* samples = 73) and an AraGene microarray set[19] (*N* samples = 72). Methods using within-study-normalization (WSN) listed. Boxplot properties include: centre line = median-absolute-error (MdAE), orange box limits = interquartile range (IQR), whiskers = 1.5 x IQR. Comparisons made using a two-tailed Wilcoxon signed-rank test, with Bonferroni adjustment of P-values. n.s no significance, * P < 0.05, ** P < 0.01, *** P < 0.001, **** P < 0.0001. Figure source data with actual P-values provided in Source Data file.

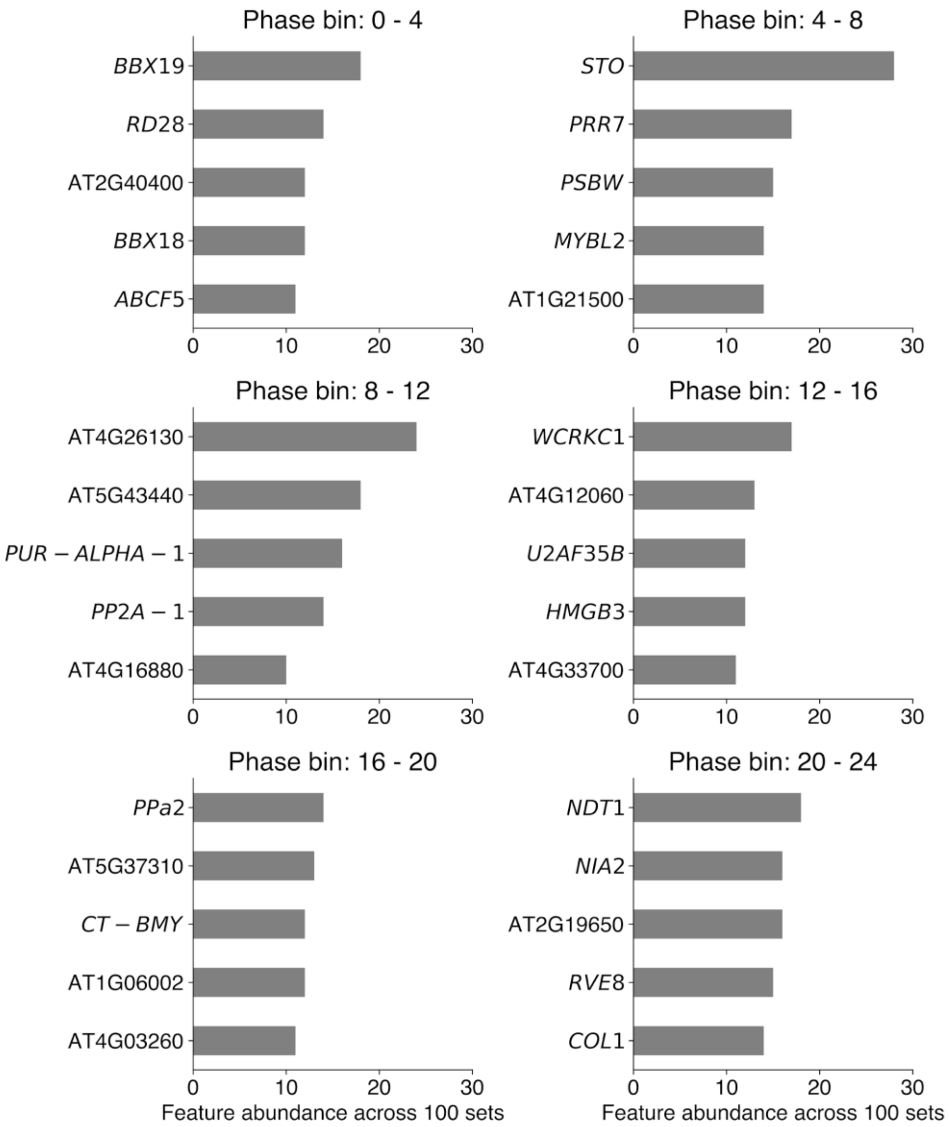

Supplementary Figure 10: Gene counts across the 100 feature sets of the ChronoGauge ensemble including the top 5 abundant genes from each phase bin including bins with phases ranging 0-4, 4-8, 8-12, 12-16, 16-20 and 20-24) used within the sequential feature selection (SFS) algorithm. Figure source data provided in Source Data file.

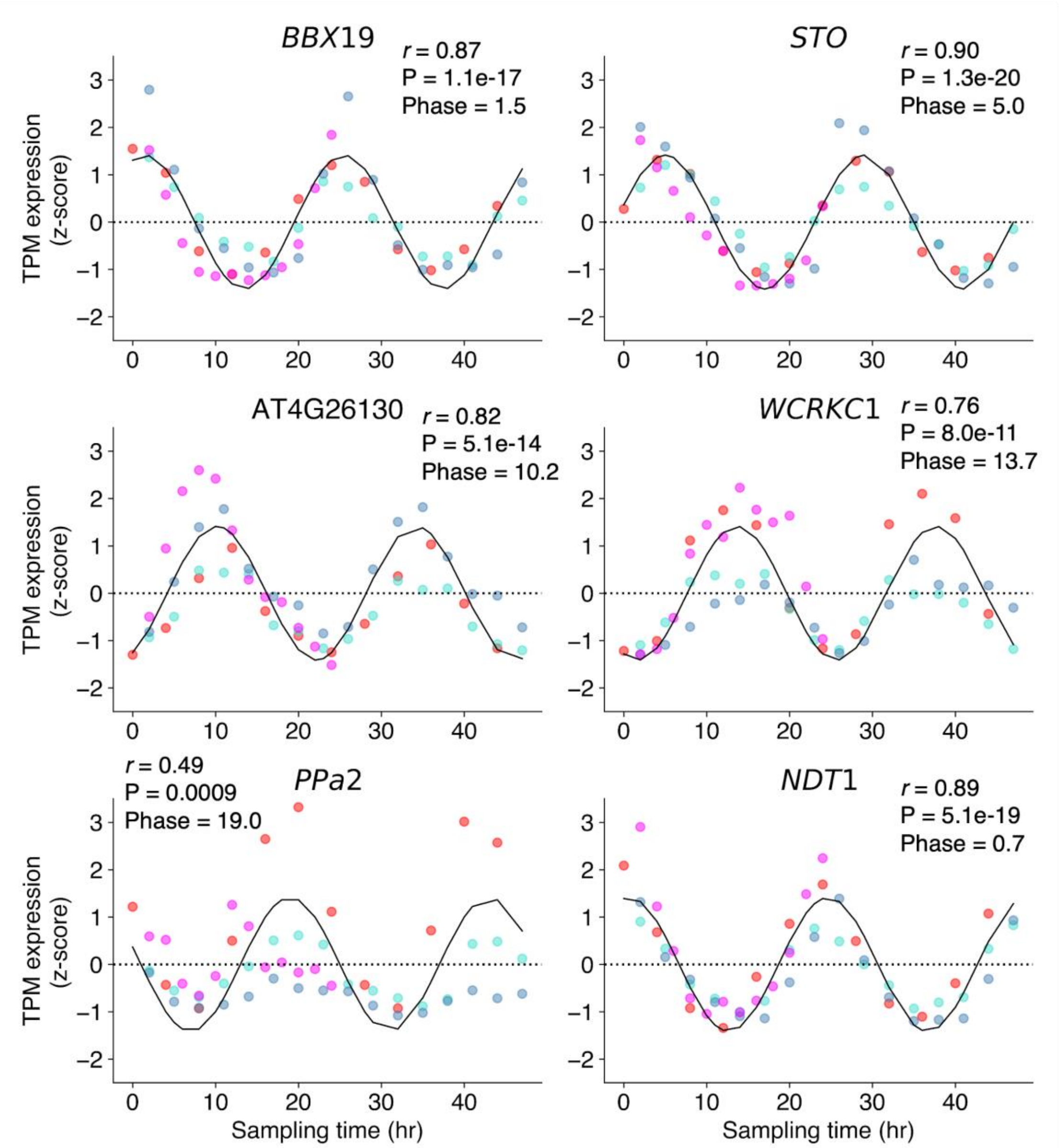

Supplementary Figure 11: Comparison of z-score scaled expression patterns across the training datasets[2,22,23] corresponding to the genes giving the top counts across the 100 feature sets of the ChronoGauge ensemble. Expression values included for all 4 training datasets. Curves show the optimal cosine wave fitted using the MolecularTimetable[6]-based iterative approach. Pearson correlation coefficients (*r*), Bonferroni-adjusted P-values and approximated phase listed. Figure source data provided in Source Data file.

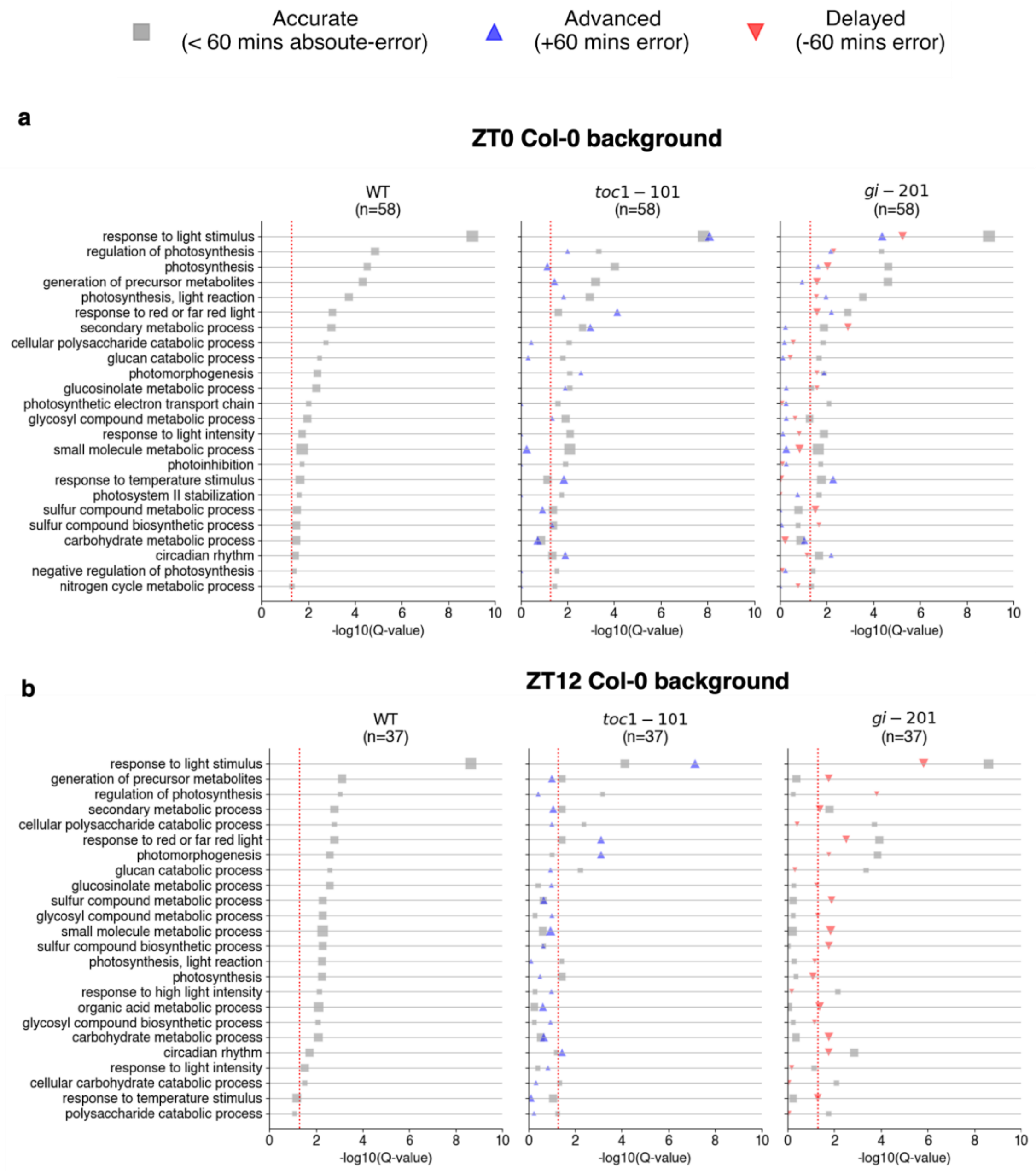

Supplementary Figure 12: Go term enrichment of biological processes across wild-type (WT) and knock-out mutants *toc1-101 and gi-201* within the *Graf et al.*[12] dataset. Col-0 *Arabidopsis* samples harvested at **a** ZT0 and **b** ZT12. Significance determined using Fisher's test in TopGO[24] with Benjamini-Hochberg adjusted P-values (Q-values) and a threshold of Q < 0.05 (red line). Figure source data provided in Source Data file.

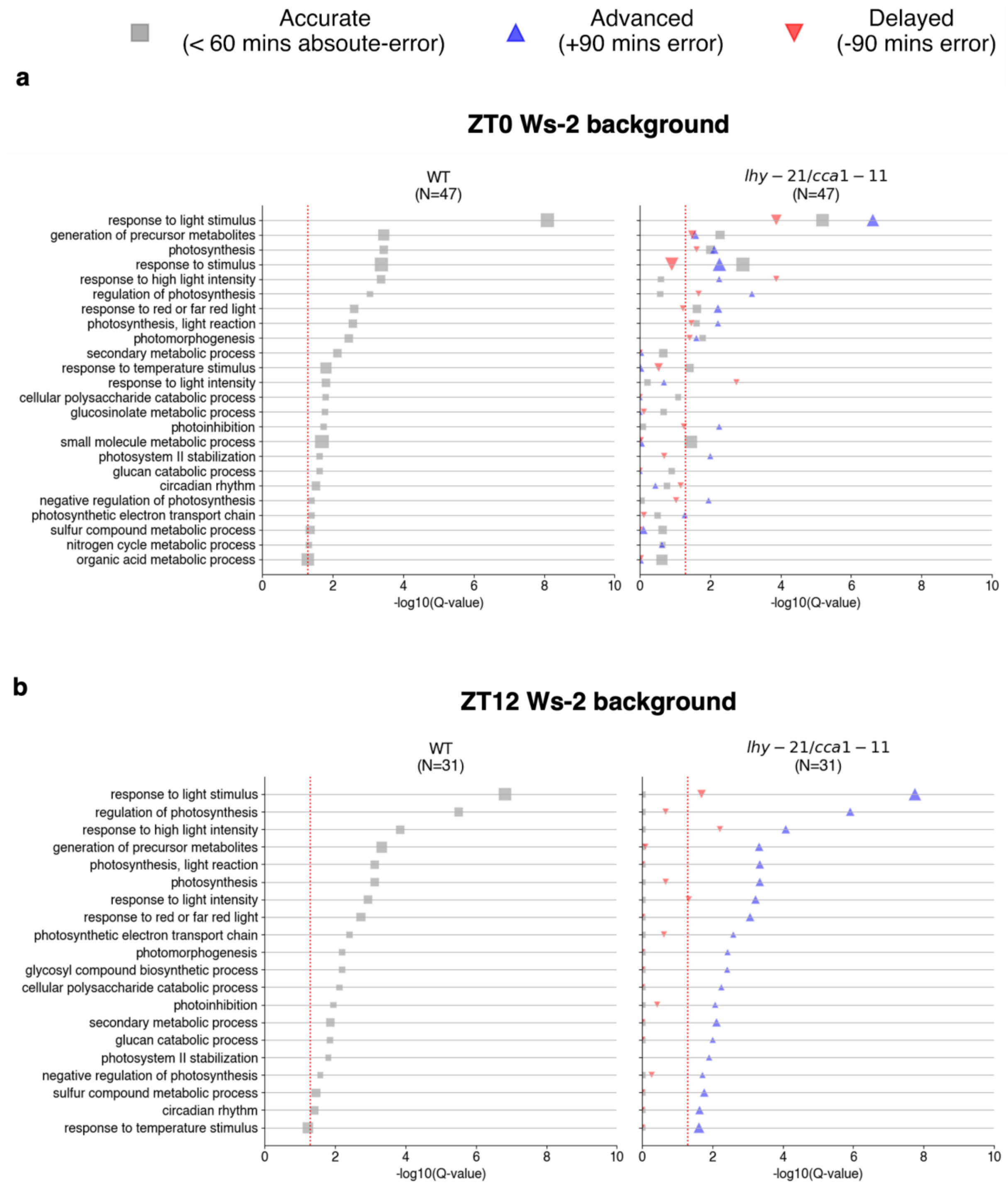

Supplementary Figure 13: Go term enrichment of biological processes across wild-type (WT) plants and knock-out mutants *lhy-21/cca1-11* within the *Graf et al.*[12] dataset. Ws-2 *Arabidopsis* samples harvested at **a** ZT0 and **b** ZT12. Significance determined using Fisher's test in TopGO[24] with Benjamini-Hochberg adjusted P-values (Q-value) and a threshold of Q < 0.05 (red line). Figure source data provided in Source Data file.

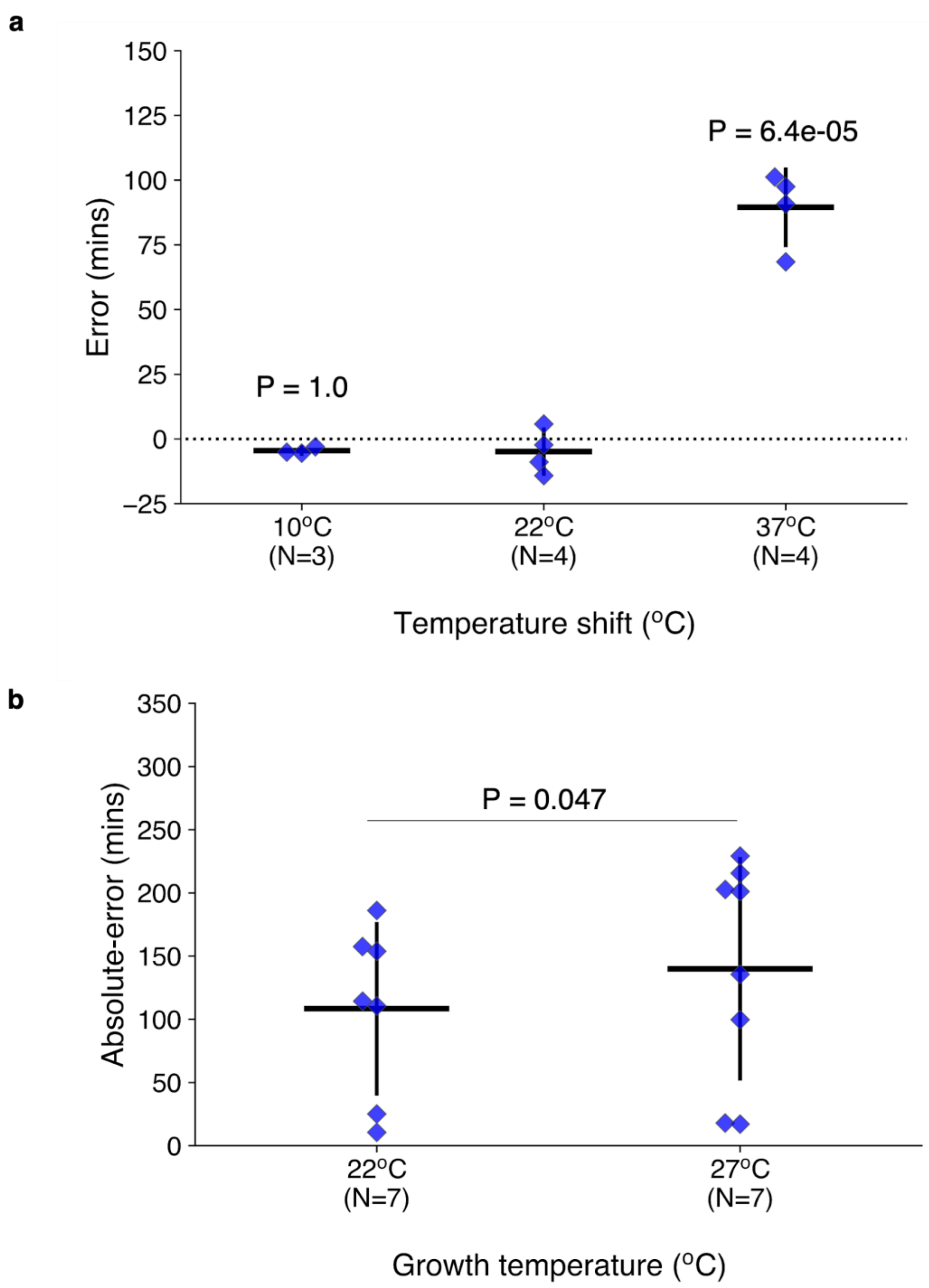

Supplementary Figure 14: Comparison of circadian time (CT) estimates in response to different temperature conditions. Includes **a** difference in CT errors at ZT1 after a temperature shift from 22°C to 10°C or 37°C one hour prior to sampling within *Blair et al*.[25] dataset. Significance against the control kept at 22°C determined using a two-tailed independent T-test P-values listed with correction using Bonferroni adjustment. **b** Comparison of absolute errors of CT estimates made across a time-course where plants were harvested or grown at either 22°C or 27°C within *Ezer et al.*[11] dataset. Significance determined using a Wilcoxon signed-rank test with Bonferroni adjustment of P-values. Both strip plot properties include: centre line = mean, box limits = interquartile range (IQR), whiskers = 1.5 x IQR, blue diamonds = error of individual samples. Figure source data provided in Source Data file.

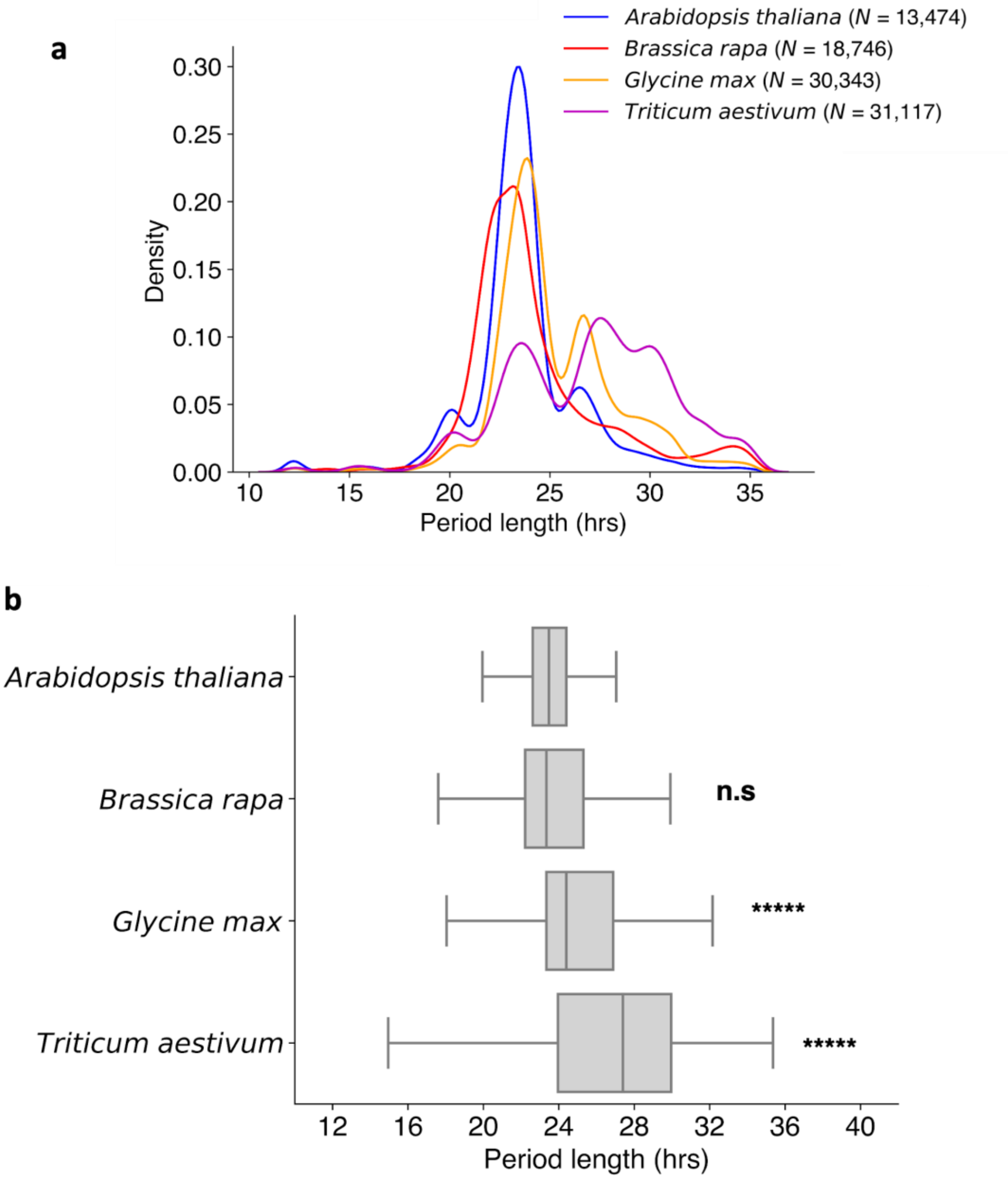

Supplementary Figure 15: Period length (meta2d, hrs) approximations found using Metacycle[1] for the expression of genes determined to be circadian regulated (meta2d Q < 0.05) across continuous-light (LL) time-courses for different species including *Arabidopsis thaliana* (based on the *Romanowski et al.*[2] time-course; *N* circadian regulated genes = 13,474), *Brassica rapa*[26] (*N* = 18,746), *Glycine max*[27] (*N* = 30,343) and *Triticum aestivum*[28] (*N* = 31,117). Includes **a** distribution of period lengths across each species and **b** comparison of period lengths between *A. thaliana* and other species. Comparison made using Two-tailed Mann-Whitney U test with Bonferroni adjustment of P-values. Figure source data with actual P-values provided in Source Data file.

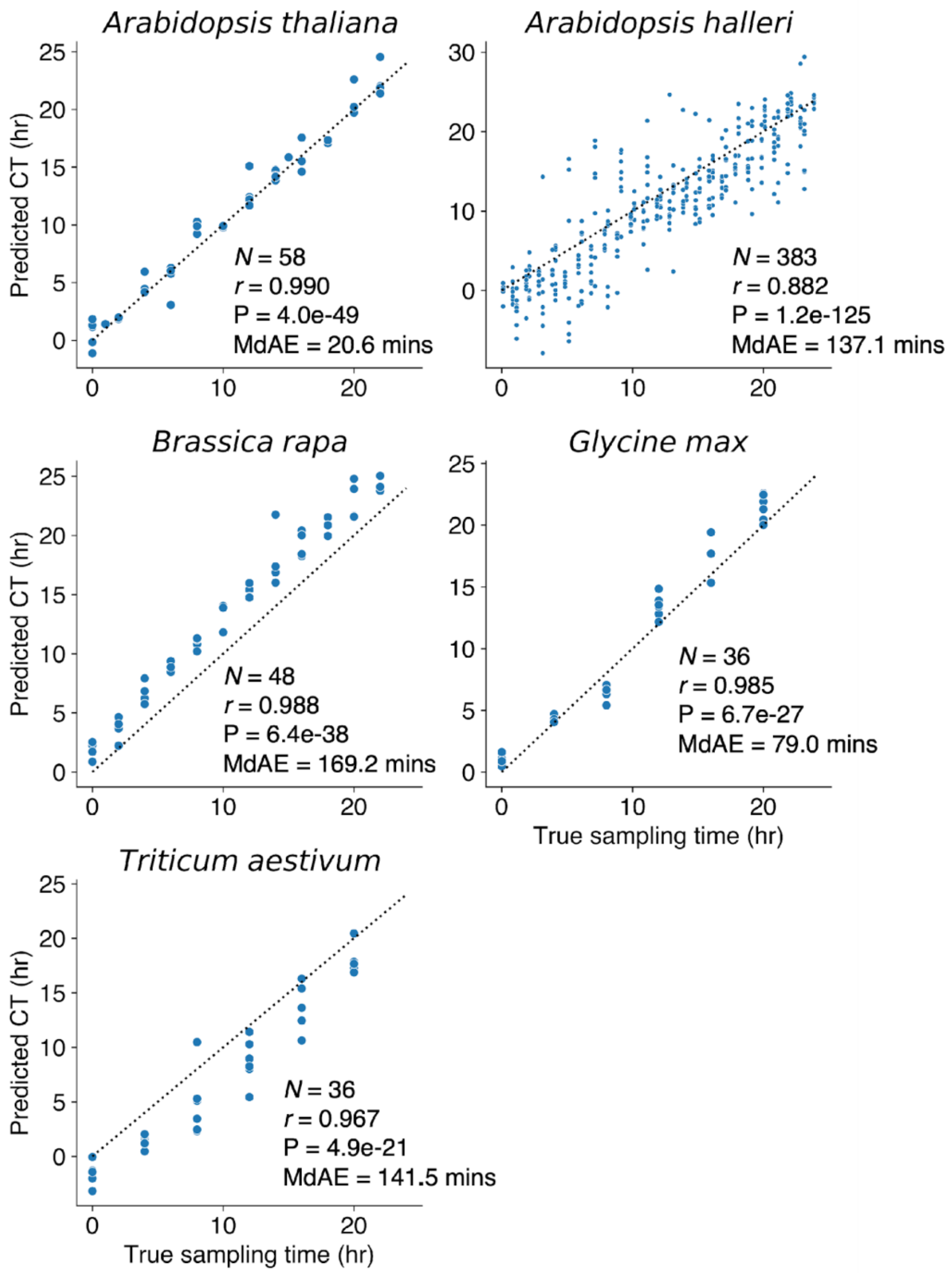

Supplementary Figure 16: Correlation of circadian time (CT) estimates with true sampling times across RNA-seq validation sets for *A. thaliana*[8–13] and non-model species using ChronoGauge fit only to *A. thaliana* training data[2,22,23]. For non-model species, ortholog genes were found that mapped to each of the ChronoGauge ensemble sub-predictor's *A. thaliana* gene features. Non-model species include *A. halleri*[29,30] (harvested from natural environments), *B. rapa*[26], *G. max*[27] and *T. aestivum*[28]. Pearson correlation coefficients (*r*) listed alongside Bonferroni adjusted P-values and median-absolute-errors (MdAEs). Figure source data provided in Source Data file.

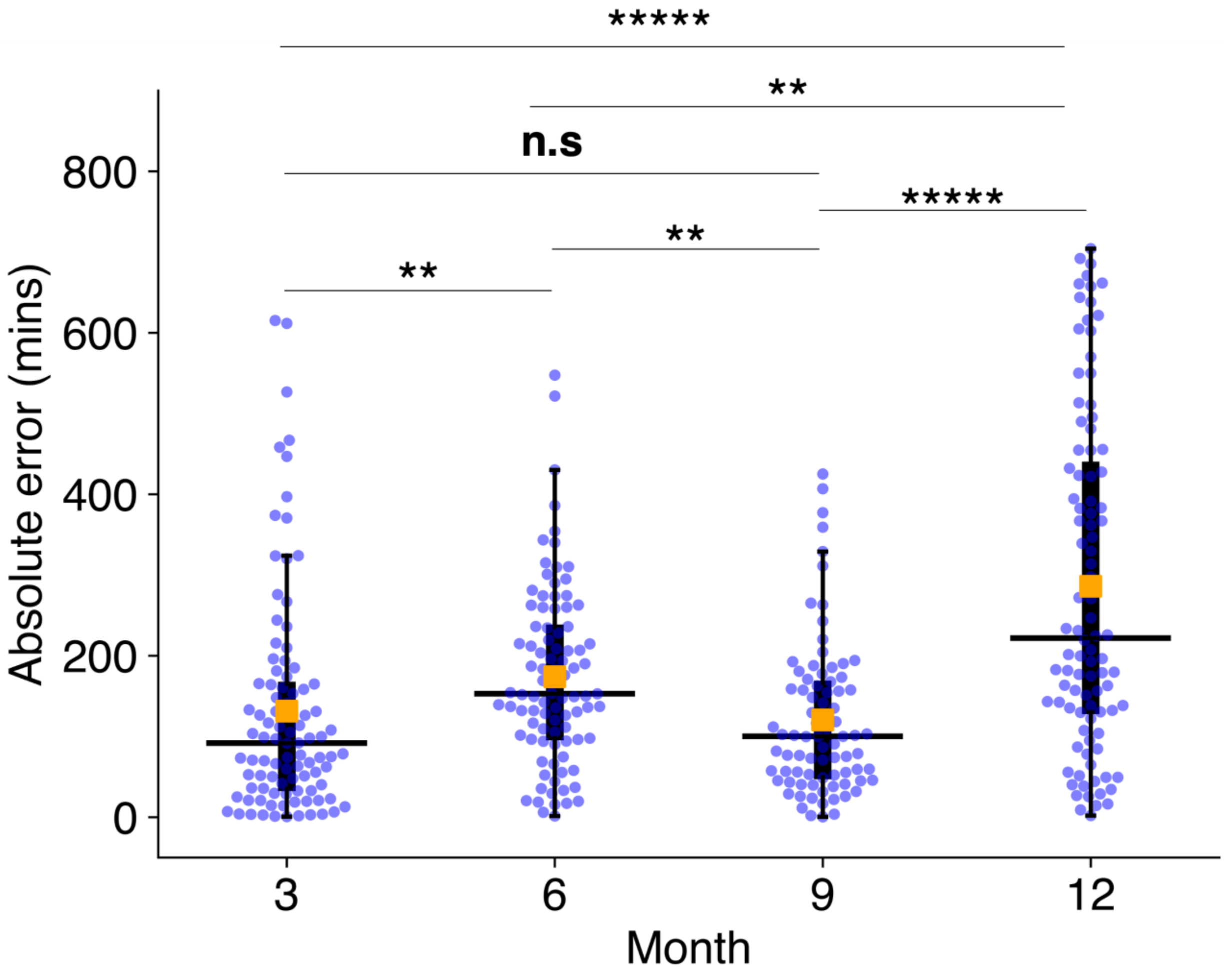

Supplementary Figure 17: Comparison of absolute errors of circadian time (CT) estimates made across *Arabodopsis halleri* samples from natural conditions[29,30] with associated weather data ($N = 367$). Boxplot properties include: centre line = median-absolute-error (MdAE), orange box = mean-absolute-error (MAE), box limits = interquartile range (IQR), whiskers = 1.5 x IQR, blue points = error of individual samples. Absolute errors compared using a Two-tailed Mann-Whitney U test with Bonferroni adjustment of P-values. n.s no significance, ** P < 0.01, ***** P < 0.00001. Figure source data and actual P-values provided in Source Data file.

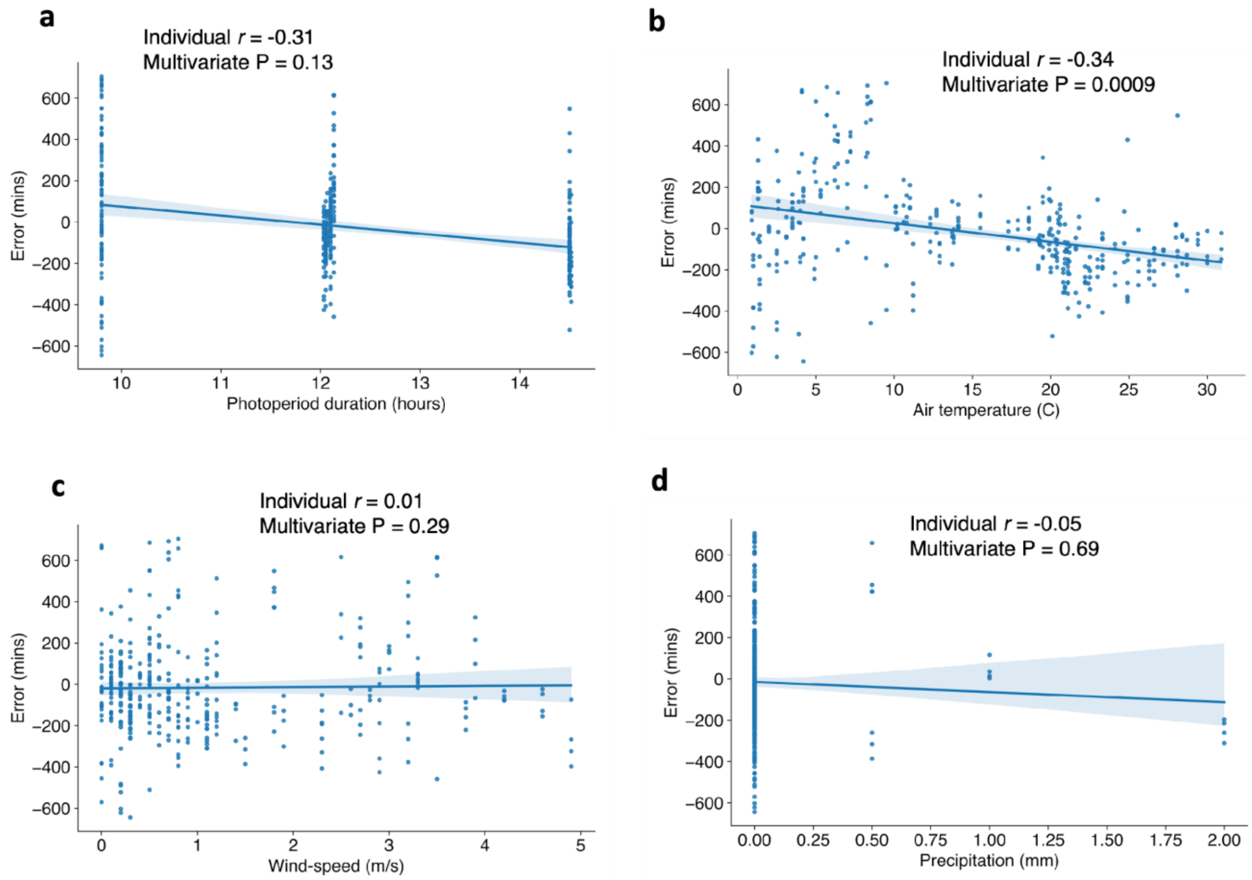

Supplementary Figure 18: Correlation of wild *Arabidopsis halleri* sample[29,30] circadian time (CT) estimate errors ($N = 367$) with available environmental meta-data including **a** the photoperiod duration (dusk – dawn, hours), **b** air temperature (ºC), **c** wind-speed (m/s) and **d** the precipitation (mm) at sampling. Individual Pearson correlation coefficients (*r)* listed. Additionally, a multivariate ordinary-least-squares regression model was used to test the significance of the association while accounting for confounding environmental variables, with multivariate P-values being listed. Figure source data provided in Source Data file.

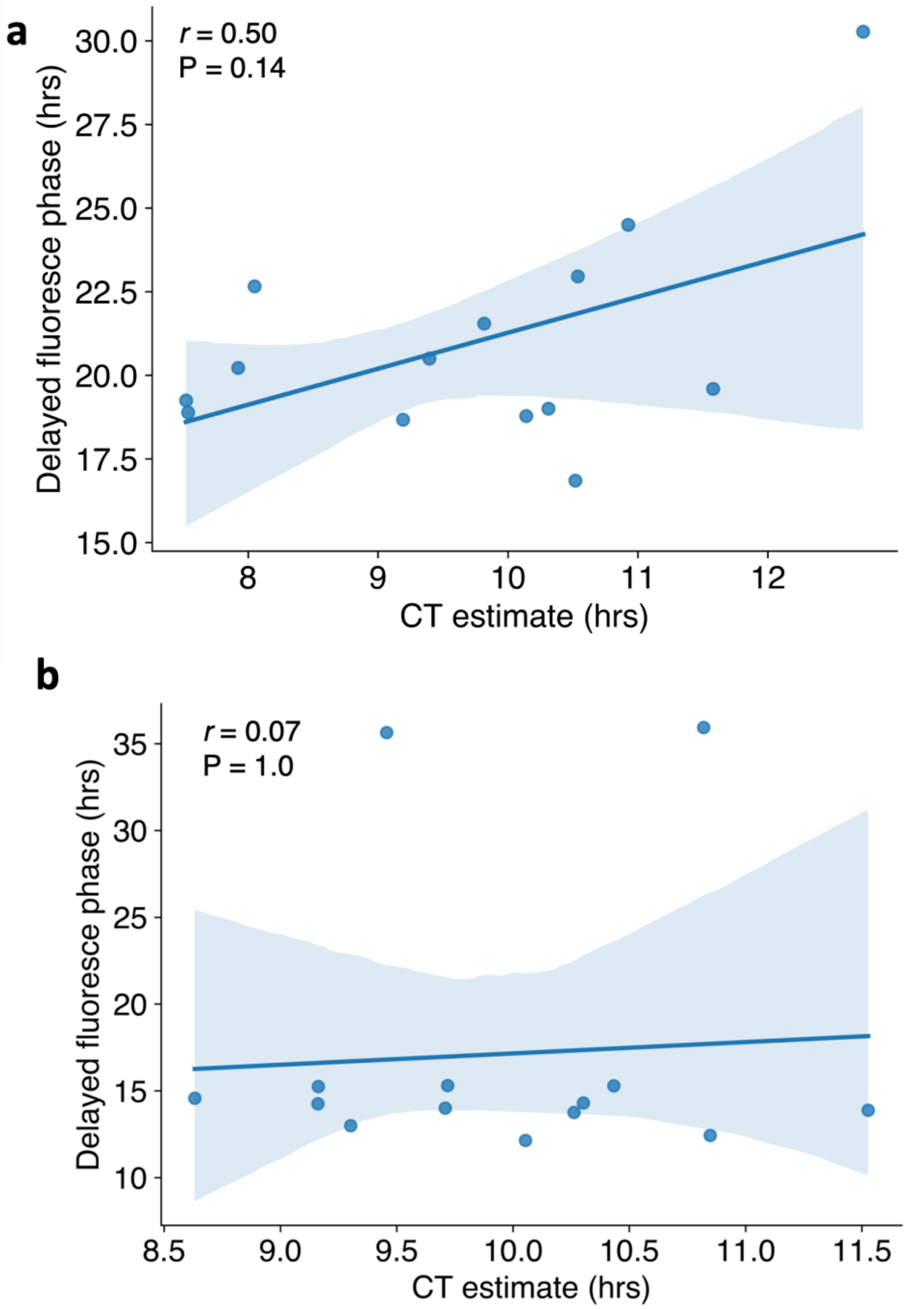

Supplementary Figure 19: Correlation of circadian time (CT) estimates made for *Arabidopsis* accessions (*N* = 14) using RNA-seq data by *Dubin et al*.[31] with phase approximations of the same accessions made using delayed fluorescence by *Rees et al.*[32] Includes **a** plants grown at 10ºC and **b** plants grown at 16ºC. Pearson correlation coefficient (*r*) listed with Bonferroni adjusted P-values. Figure source data provided in Source Data file.

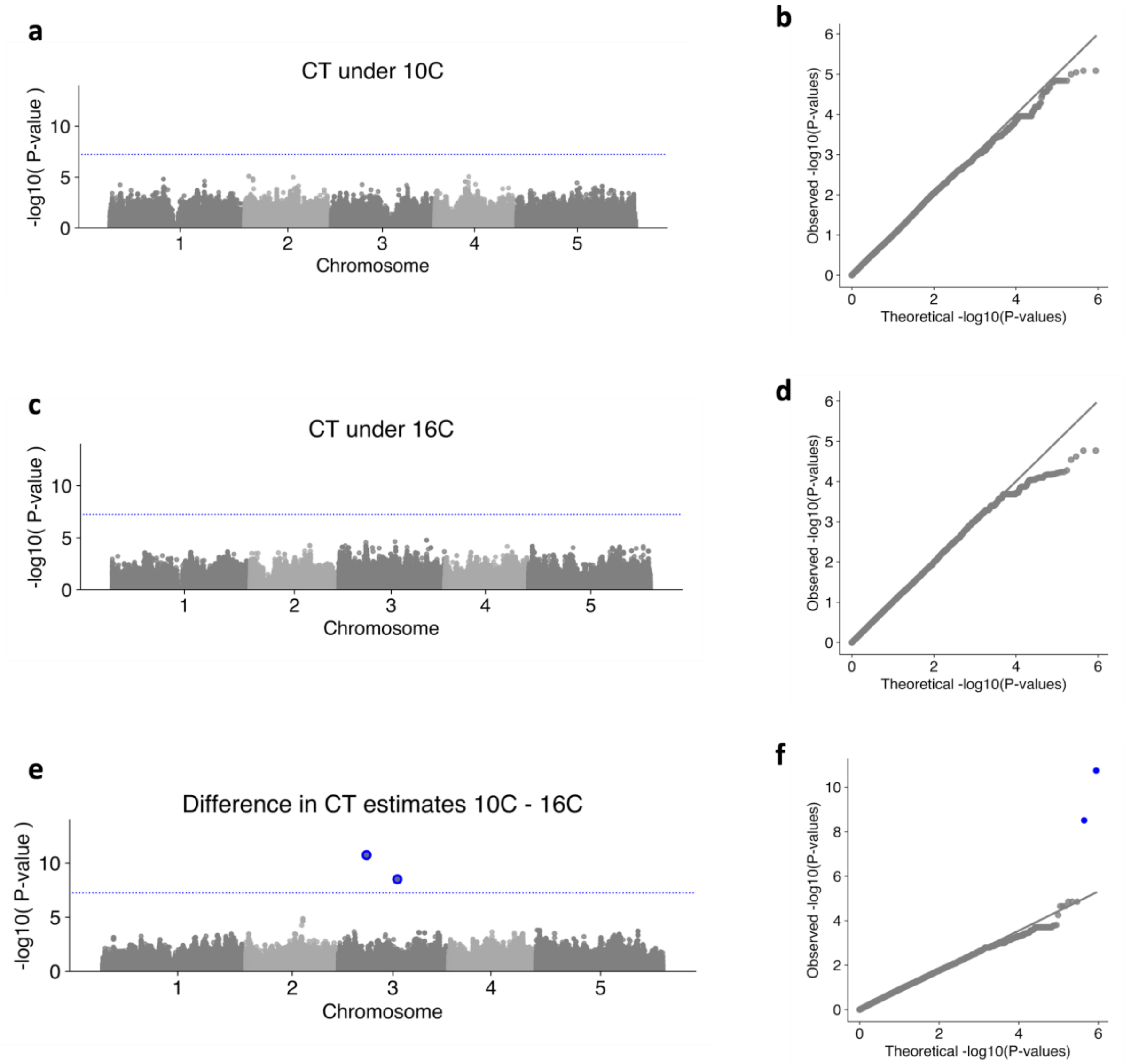

Supplementary Figure 20: Identification of marker-trait-associations (MTAs) across 153 *Arabidopsis* accessions within the *Dubin et al.*[31]. RNA-seq dataset using phenotypes based on circadian time (CT) estimates for each accession within the genome-wide-association-study model BLINK[33] within the R package GAPIT[34]. Genotype information for the accessions were acquired from the 1001 Genomes Project[35]. Includes significance of each single-nucleotide-polymorphism (SNP) site's association with and quantile-quantile plots using the phenotypes **a,b** CT estimates in samples grown under 10ºC, **c,d** CT estimates in samples grown under 16ºC and **e,f** difference between CT estimates grown under the two temperature groups (10ºC – 16ºC) respectively. Analyses included $N = 880{,}417$ SNP sites. Significant SNPs shown (blue circle) based on Bonferroni adjustment of P-values within each study. Adj. $P < 0.05$ corresponds to unadjusted $P < 5.7e{-}08$ (blue line). Source data for figures provided within an Open Science Framework (OSF) file[36].

References

1. Wu, G., Anafi, R. C., Hughes, M. E., Kornacker, K. & Hogenesch, J. B. MetaCycle: an integrated R package to evaluate periodicity in large scale data. *Bioinformatics* **32**, 3351–3353 (2016).
2. Romanowski, A., Schlaen, R. G., Perez-Santangelo, S., Mancini, E. & Yanovsky, M. J. Global transcriptome analysis reveals circadian control of splicing events in Arabidopsis thaliana. *Plant J.* **103**, 889–902 (2020).
3. Hughey, J. J., Hastie, T. & Butte, A. J. ZeitZeiger: supervised learning for high-dimensional data from an oscillatory system. *Nucleic Acids Res.* **44**, e80 (2016).
4. Laing, E. E. *et al.* Blood transcriptome based biomarkers for human circadian phase. *eLife* **6**, e20214 (2017).
5. Duan, J. *et al.* tauFisher predicts circadian time from a single sample of bulk and single-cell pseudobulk transcriptomic data. *Nat. Commun.* **15**, 3840 (2024).
6. Ueda, H. R. *et al.* Molecular-timetable methods for detection of body time and rhythm disorders from single-time-point genome-wide expression profiles. *Proc. Natl. Acad. Sci.* **101**, 11227–11232 (2004).
7. Braun, R. *et al.* Universal method for robust detection of circadian state from gene expression. *Proc. Natl. Acad. Sci. U. S. A.* **115**, E9247–E9256 (2018).
8. Rugnone, M. L. *et al.* LNK genes integrate light and clock signaling networks at the core of the Arabidopsis oscillator. *Proc. Natl. Acad. Sci. U. S. A.* **110**, 12120 (2013).
9. Miller, M., Song, Q., Shi, X., Juenger, T. E. & Chen, Z. J. Natural variation in timing of stress-responsive gene expression predicts heterosis in intraspecific hybrids of Arabidopsis. *Nat. Commun.* **6**, 7453 (2015).
10. Takahashi, N., Hirata, Y., Aihara, K. & Mas, P. A Hierarchical Multi-oscillator Network Orchestrates the Arabidopsis Circadian System. *Cell* **163**, 148–159 (2015).
11. Ezer, D. *et al.* The Evening Complex coordinates environmental and endogenous signals in Arabidopsis. *Nat. Plants* **3**, 17087 (2017).
12. Graf, A. *et al.* Parallel analysis of Arabidopsis circadian clock mutants reveals different scales of transcriptome and proteome regulation. *Open Biol.* **7**, 160333 (2017).

13. Dubois, M., Claeys, H., Van den Broeck, L. & Inzé, D. Time of day determines Arabidopsis transcriptome and growth dynamics under mild drought. *Plant Cell Environ.* **40**, 180–189 (2017).
14. Zhang, Y., Parmigiani, G. & Johnson, W. E. ComBat-seq: batch effect adjustment for RNA-seq count data. *NAR Genomics Bioinforma.* **2**, lqaa078 (2020).
15. Edwards, K. D. *et al.* FLOWERING LOCUS C Mediates Natural Variation in the High-Temperature Response of the Arabidopsis Circadian Clock. *Plant Cell* **18**, 639–650 (2006).
16. Covington, M. F. & Harmer, S. L. The Circadian Clock Regulates Auxin Signaling and Responses in Arabidopsis. *PLoS Biol.* **5**, e222 (2007).
17. Michael, T. P. *et al.* A Morning-Specific Phytohormone Gene Expression Program underlying Rhythmic Plant Growth. *PLOS Biol.* **6**, e225 (2008).
18. Espinoza, C. *et al.* Interaction with Diurnal and Circadian Regulation Results in Dynamic Metabolic and Transcriptional Changes during Cold Acclimation in Arabidopsis. *PLOS ONE* **5**, e14101 (2010).
19. Endo, M., Shimizu, H., Nohales, M. A., Araki, T. & Kay, S. A. Tissue-specific clocks in Arabidopsis show asymmetric coupling. *Nature* **515**, 419–422 (2014).
20. Gardiner, L.-J. *et al.* Interpreting machine learning models to investigate circadian regulation and facilitate exploration of clock function. *Proc. Natl. Acad. Sci. U. S. A.* **118**, e2103070118 (2021).
21. Takeoka, M. *et al.* Estimation of the Circadian Phase by Oscillatory Analysis of the Transcriptome in Plants. *Environ. Control Biol.* **56**, 67–72 (2018).
22. Cortijo, S., Aydin, Z., Ahnert, S. & Locke, J. C. Widespread inter-individual gene expression variability in Arabidopsis thaliana. *Mol. Syst. Biol.* **15**, (2019).
23. Yang, Y., Li, Y., Sancar, A. & Oztas, O. The circadian clock shapes the Arabidopsis transcriptome by regulating alternative splicing and alternative polyadenylation. *J. Biol. Chem.* **295**, 7608 (2020).
24. topGO. *Bioconductor* http://bioconductor.org/packages/topGO/.
25. Blair, E. J. *et al.* Contribution of time of day and the circadian clock to the heat stress responsive transcriptome in Arabidopsis. *Sci. Rep.* **9**, 4814 (2019).

26. Greenham, K. *et al.* Expansion of the circadian transcriptome in Brassica rapa and genome-wide diversification of paralog expression patterns. *eLife* **9**, e58993 (2020).

27. Li, M. *et al.* Comprehensive mapping of abiotic stress inputs into the soybean circadian clock. *Proc. Natl. Acad. Sci.* **116**, 23840–23849 (2019).

28. Rees, H. *et al.* Circadian regulation of the transcriptome in a complex polyploid crop. *PLOS Biol.* **20**, e3001802 (2022).

29. Nagano, A. J. *et al.* Annual transcriptome dynamics in natural environments reveals plant seasonal adaptation. *Nat. Plants* **5**, 74–83 (2019).

30. Honjo, M. N. *et al.* Seasonality of interactions between a plant virus and its host during persistent infection in a natural environment. *ISME J.* **14**, 506–518 (2020).

31. Dubin, M. J. *et al.* DNA methylation in Arabidopsis has a genetic basis and shows evidence of local adaptation. *eLife* **4**, e05255 (2015).

32. Rees, H., Joynson, R., Brown, J. K. M. & Hall, A. Naturally occurring circadian rhythm variation associated with clock gene loci in Swedish Arabidopsis accessions. *Plant Cell Environ.* **44**, 807–820 (2021).

33. Huang, M., Liu, X., Zhou, Y., Summers, R. M. & Zhang, Z. BLINK: a package for the next level of genome-wide association studies with both individuals and markers in the millions. *GigaScience* **8**, (2019).

34. Wang, J. & Zhang, Z. GAPIT Version 3: Boosting Power and Accuracy for Genomic Association and Prediction. *Genomics Proteomics Bioinformatics* **19**, 629 (2021).

35. Alonso-Blanco, C. *et al.* 1,135 Genomes Reveal the Global Pattern of Polymorphism in Arabidopsis thaliana. *Cell* **166**, 481–491 (2016).

36. Reynolds, C. ChronoGauge paper data & code. *Open Science Framework* (2024). https://doi.org/10.17605/OSF.IO/839EM
