# Supplementary material for: Machine learning models highlight environmental and genetic factors associated with the Arabidopsis circadian clock
Source: Nat Commun. 2025 Aug 5;16:7223. doi: 10.1038/s41467-025-62196-w (PMC12325936; doi:10.1038/s41467-025-62196-w)
Supplement: Supplementary file 2 — Description of Additional Supplementary Files [file 41467_2025_62196_MOESM2_ESM.pdf]

Description of Additional Supplementary Files

Supplementary Data 1

Description of *Arabidopsis* RNA-seq time-series datasets which were combined and used for training ChronoGauge and other circadian time (CT) predictors. All CT predictions in this work were made after fitting models to these datasets. Includes accession numbers, authors and associated meta-data. Appraisal and cross-validation results across these combined training datasets are shown in Supplementary Figures 2–4.

Supplementary Data 2

Circadian summary statistics produced for each expressed gene by MetaCycle describing rhythmic parameter approximations and P-values within the continuous-light time-series experiment by *Romanowski et al.*. Includes results from ARS and JTK_CYCLE methods, in addition to the meta2d aggregation of results from these two methods. Meta2d Q-values and phases specifically were used in the sequential feature selection method described in Figure 1.

Supplementary Data 3

Description of *Arabidopsis* RNA-seq and microarray samples used for benchmarking ChronoGauge predictions against other models. Includes accession numbers, authors and associated meta-data. Predictions made for these datasets are shown in Figure 2b, Figure 3a and Supplementary Figures 5-9.

Supplementary Data 4

Description of *Arabidopsis* RNA-seq samples used for testing hypotheses related to experimental conditions including mutations of clock genes and exposures to different temperatures. Includes accession numbers, authors and associated meta-data. Predictions made for these datasets are shown in Figure 4 and Figure 6, and Supplementary Figure 14 and Supplementary Figures 18-19.

Supplementary Data 5

Description of RNA-seq time-series datasets which do not belong to *A. thaliana* which were used to test ChronoGauge's application in non-model plant species. Species listed include continuous-light data in *Triticum aestevum* (wheat), *Brassica rapa* and *Glycine max* (soybean), in addition to *Arabidopsis helleri* samples which were harvested from natural conditions. Includes accession numbers, authors and associated meta-data. Predictions made for this dataset are shown in Figure 5 and Supplementary Figures 15 – 18.
